# Supplementary material for: C-Reactive Protein to Albumin Ratio as Prognostic Marker in Locally Advanced Non-Small Cell Lung Cancer Treated with Chemoradiotherapy
Source: Biomedicines. 2022 Mar 3;10(3):598. doi: 10.3390/biomedicines10030598 (PMC8945805; doi:10.3390/biomedicines10030598)
Supplement: Supplementary file 1 [file biomedicines-10-00598-s001.zip › biomedicines-1617987-supplementary.pdf]

biomedicines

MDPI

Article

C-Reactive Protein to Albumin Ratio as Prognostic Marker in Locally Advanced Non-Small Cell Lung Cancer Treated with Chemoradiotherapy

Supplementary Material

Supplementary Table S1. Univariate Cox regression analysis.

		HR	95% CI	*p*-value
**Total cohort**				
LPFS	Sex	2.711	0.979-7.508	**0.046**
	Leukocytes nadir	0.257	0.084-0.793	**0.011**
**Adenocarcinomas and squamous cell carcinomas**				
OS	CAR	2.268	1.086-4.736	**0.025**
	BMI	2.161	1.014-4.604	**0.041**
	Histology	2.456	1.178-5.122	**0.013**
PFS	CAR	2.105	1.031-4.295	**0.037**
LPFS	CAR	4.426	1.493-13.121	**0.004**
	Sex	3.303	1.097-9.943	**0.025**
	Histology	3.826	1.304-11.225	**0.009**
	Leukocytes nadir	0.200	0.056-0.712	**0.006**
DMFS	CAR	1.530	0.656-3.569	0.322

Abbreviations: LPFS, local progression-free survival; OS, overall survival; CAR, C-reactive protein to albumin ratio; BMI, body mass index; PFS, progression-free survival; DMFS, distant metastasis-free survival.

Supplementary Table S2. Univariate Cox regression analysis for all variables (total cohort).

		HR	95% CI	*p*-value
OS	Sex	1.608	0.806-3.207	0.174
	T-stage	0.636	0.323-1.254	0.188
	N-stage	0.621	0.254-1.518	0.292
	ECOG	0.918	0.453-1.859	0.813
	CT prior to CRT	1.709	0.521-2.235	0.838
	Definitive vs. neoadjuvant	0.869	0.356-2.124	0.758
	BMI	1.852	0.930-3.688	0.075
	Histology	-	-	-
	Thrombocytes baseline	1.088	0.505-2.343	0.830
	Thrombocytes nadir	0.621	0.309-1.213	0.156
	Hemoglobin baseline	1.302	0.660-2.568	0.445
	Hemoglobin nadir	0.683	0.344-1.356	0.273
	Leukocytes baseline	0.777	0.367-1.648	0.510
	Leukocytes nadir	0.700	0.351-1.396	0.309
PFS	Sex	1.603	0.827-3.107	0.159
	T-stage	1.086	0.548-2.152	0.813
	N-stage	0.537	0.219-1.316	0.167

	ECOG	1.353	0.645-2.836	0.422
	CT prior to CRT	1.045	0.501-2.178	0.907
	Definitive vs. neoadjuvant	0.780	0.300-2.029	0.609
	BMI	1.583	0.820-3.059	0.168
	Histology	-	-	-
	Thrombocytes baseline	1.341	0.645-2.787	0.431
	Thrombocytes nadir	0.719	0.372-1.392	0.326
	Hemoglobin baseline	1.438	0.740-2.796	0.282
	Hemoglobin nadir	0.850	0.439-1.646	0.630
	Leukocytes baseline	0.753	0.364-1.560	0.444
	Leukocytes nadir	1.132	0.584-2.194	0.713
LPFS	Sex	2.711	0.979-7.508	**0.046**
	T-stage	1.065	0.385-2.941	0.904
	N-stage	0.384	0.104-1.422	0.137
	ECOG	2.556	0.724-9.022	0.131
	CT prior to CRT	0.808	0.257-2.537	0.714
	Definitive vs. neoadjuvant	0.356	0.047-2.705	0.297
	BMI	1.146	0.441-2.978	0.780
	Histology	-	-	-
	Thrombocytes baseline	1.580	0.569-4.387	0.376
	Thrombocytes nadir	0.467	0.169-1.292	0.314
	Hemoglobin baseline	1.924	0.708-5.228	0.192
	Hemoglobin nadir	0.474	0.174-1.289	0.135
	Leukocytes baseline	0.595	0.189-1.872	0.370
	Leukocytes nadir	0.257	0.084-0.793	**0.011**
DMFS	Sex	1.591	0.721-3.510	0.246
	T-stage	0.734	0.332-1.624	0.444
	N-stage	0.137	0.338-3.820	0.835
	ECOG	1.053	0.449-2.468	0.905
	CT prior to CRT	1.522	0.669-3.459	0.313
	Definitive vs. neoadjuvant	0.855	0.289-2.528	0.777
	BMI	1.635	0.742-3.603	0.218
	Histology	-	-	-
	Thrombocytes baseline	1.439	0.599-3.458	0.414
	Thrombocytes nadir	0.871	0.395-1.922	0.733
	Hemoglobin baseline	1.443	0.647-3.219	0.368
	Hemoglobin nadir	1.008	0.458-2.218	0.984
	Leukocytes baseline	0.743	0.304-1.814	0.513
	Leukocytes nadir	1.266	0.574-2.797	0.558

Differences in histology were only calculated between adenocarcinoma and squamous cell carcinoma (non-squamous cell carcinomas and non-adenocarcinomas excludes), as the results would be distorted due to the small number of different histological subtypes. Abbreviations: CRT, chemoradiotherapy; CT, chemotherapy; BMI, body mass index; OS, overall survival; DFS, disease-free survival; LPFS, local progression-free survival; DMFS, distant metastasis-free survival.

Supplementary Table S3. Univariate Cox regression analysis with GPS.

		HR	95% CI	*p*-value
OS	GPS 0			
	GPS 1	0.719	0.160-3.222	
	GPS 2	1.145	0.266-4.933	
				0.441
PFS	GPS 0			
	GPS 1	0.711	0.202-2.501	
	GPS 2	0.983	0.287-3.367	
				0.634
LPFS	GPS 0			
	GPS 1	0.626	0.073-5.374	
	GPS 2	1.556	0.199-12.185	
				0.222
DMFS	GPS 0			
	GPS 1	1.207	0.153-9.523	
	GPS 2	1.537	0.200-11.804	
				0.800

Abbreviations: GPS, Glasgow Prognostic Score; OS, overall survival; PFS, progression-free survival; LPFS, local progression-free survival; DMFS, distant metastasis-free survival.

Supplementary Table S4. Multivariate Cox regression analysis for adenocarcinomas or squamous cell carcinomas only.

		HR	95% CI	*p*-value
OS	CAR	2.480	1.178-5.220	**0.018**
	BMI	2.359	1.103-5.045	**0.024**
	Histology	removed		
LPFS	CAR	3.031	0.927-9.911	0.061
	Leukocytes nadir	0.198	0.052-0.758	**0.009**
	Sex	2.987	0.900-9.919	0.065
	Histology	3.011	0.916-9.891	0.066

Abbreviations: OS, overall survival; CAR, C-reactive protein to albumin ratio; BMI, body mass index; LPFS, local progression-free survival.
